# The Role of Speckle Tracking Echocardiography in the Evaluation of Advanced-Heart-Failure Patients

**DOI:** 10.3390/jcm13144037

**Published:** 2024-07-10

**Authors:** Luca Martini, Matteo Lisi, Maria Concetta Pastore, Francesca Maria Righini, Andrea Rubboli, Michael Y. Henein, Matteo Cameli

**Affiliations:** 1 Department of Medical Biotechnologies, Division of Cardiology, University of Siena, 53100 Siena, Italy; mariaconce.pastore@unisi.it (M.C.P.); francesca.righini@ao-siena.toscana.it (F.M.R.); cameli@unisi.it (M.C.); 2 Department of Cardiovascular Disease, AUSL Romagna, Division of Cardiology, Ospedale S. Maria delle Croci, 48121 Ravenna, Italy; matteo.lisi@auslromagna.it (M.L.); andrea.rubboli@auslromagna.it (A.R.); 3 Institute of Public Health and Clinical Medicine, Umeå University, 90187 Umeå, Sweden; michael.henein@umu.se

**Keywords:** heart failure, speckle tracking echocardiography, heart catheterization, myocardial oxygen consumption, myocardial fibrosis

## Abstract

Health care is currently showing a fall in heart failure (HF) incidence and prevalence, particularly in developed countries, but with only a subset receiving appropriate therapy to protect the heart against maladaptive processes such as fibrosis and hypertrophy. Appropriate markers of advanced HF remain unidentified, which would help in choosing the most suitable therapy and avoid major compliance problems. Speckle tracking echocardiography (STE) is a good choice, being a non-invasive imaging technique which is able to assess cardiac deformation in a variety of conditions. Several multicenter studies and meta-analyses have demonstrated the clinical application and accuracy of STE in early and late stages of HF, as well as its association with both left ventricular (LV) filling pressures and myocardial oxygen consumption. Furthermore, STE assists in assessing right ventricular free-wall longitudinal strain (RVFWLS), which is a solid predictor of right ventricle failure (RVF) following LV assist device (LVAD) implantation. However, STE is known for its limitations; despite these, it has been shown to explain symptoms and signs and also to be an accurate prognosticator. The aim of this review is to examine the advantages of STE in the early evaluation of myocardial dysfunction and its correlation with right heart catheterization (RHC) parameters, which should have significant clinical relevance in the management of HF patients.

## 1. Introduction

The European Society of Cardiology (ESC) defines heart failure (HF) as the presence of symptoms and/or signs of HF caused by a structural and/or functional abnormality of the heart, which cause high intracardiac pressures and/or insufficient cardiac output at rest and/or during exercise [1]. The 2021 ESC Guidelines categorize this pathology based on left ventricular ejection fraction (LVEF) as HF with reduced EF (HFrEF, EF ≤ 40%), mildly reduced EF (HFmrEF, EF 41–49%), or preserved EF (HFpEF, EF ≥ 50%). Despite optimum treatment, many patients progress to late-stage advanced HF [1].

Regardless of the decrease in the incidence and prevalence of HF in industrialized nations, not all patients are identified in the early stages of the disease. This inappropriate practice results in only a subset of patients who receive optimum therapy that protects their heart from maladaptive processes such as myocardial fibrosis and LV hypertrophy [2]. This under-diagnosis can be ascribed mainly to the lack of highly specific imaging markers, particularly echocardiographic ones [2]. Despite LVEF having been used as the guiding parameter for beginning advanced treatments such as Sacubitril/Valsartan and Gliflozins, cardioverter defibrillator implant (ICD) or cardiac resynchronization therapy (CRT) [1], several studies demonstrated its lower sensitivity to unmasking LV myocardial dysfunction compared to other echocardiographic parameters such as speckle tracking echocardiography (STE) [2,3]. Also, LVEF is limited by geometric assumptions, load dependency, reproducibility, and inter-observer variability, and is influenced by heart rate, rhythm disturbances and translational motion [3]. On the other hand, LV global longitudinal strain (LV GLS), has been shown to be superior to EF in several settings, such as predicting myocardial recovery and symptomatic improvement after aortic valve replacement [4], predicting all-cause and cardiovascular mortality in advanced chronic kidney disease (CKD) [5], and predicting major adverse cardiac events in HF patients [3].

Recent studies have applied STE to the left atrium (LA), based on the assumption that it is highly sensitive to pressure and volume overload because of the LA thin wall [6]. Global peak atrial longitudinal strain (PALS) has been proved the earliest parameter to alter in many conditions associated with myocardial dysfunction such as systemic hypertension and diabetes, even before the development of LV hypertrophy, reduced LVEF or LA enlargement [6,7]. Also, PALS has been shown to strongly relate to diastolic dysfunction and myocardial fibrosis (measured at histological analysis), and hence has been proposed to provide a non-invasive estimation of LV filling pressures (LVFPs) and explanations of HF symptoms and quality of life [8]. In addition to the use of STE in assessing LA function, it has been used to quantify RV longitudinal dysfunction, thus allowing evaluation of myocardial deformation using conventional two-dimensional echo images without angle dependency [9]. Indeed, several studies have demonstrated that RV free-wall longitudinal strain (RVFWLS) in advanced-HF patients strongly links with both RV stroke work index (RVSWI) and oxygen consumption (VO_2_) in end-stage HF, especially for LV assist device (LVAD) selection [10].

The aim of this review is to examine advantages of STE measurements in early evaluation of myocardial dysfunction and their correlation with right heart catheterization (RHC) parameters, in order to support the clinical application of these measurements in early- and advanced-HF patients.

## 2. Speckle Tracking Echocardiography Measurement

STE is a semi-automated and angle-independent echocardiographic technique that allows evaluation of myocardial deformation in a range of specific conditions. Using previously saved echocardiographic pictures, a specific software can differentiate each of the speckles, integrating them in functional units (kernels) that are unequivocally recognized, given their specific spatial disposition [10].

During a cardiac cycle, the computer tracks kernel movement in three spatial directions: radial, longitudinal, and circumferential. This technique allows the system to calculate deformation (strain), rate of deformation (strain rate), displacement, and rate of displacement (velocity) for the selected myocardial segments [10].

The strain (ε) can be defined as the degree of deformation (shortening) of the analyzed segment in relation to its initial dimensions. It is measured as a percentage and is expressed by the following equation:

ε=L−L0/L0

where L is the final dimension of the segment and L0 is the initial dimension. A lengthening or thickening deformation has a positive value, while a shortening or thinning deformation has a negative one [10].

**Figure 1:**
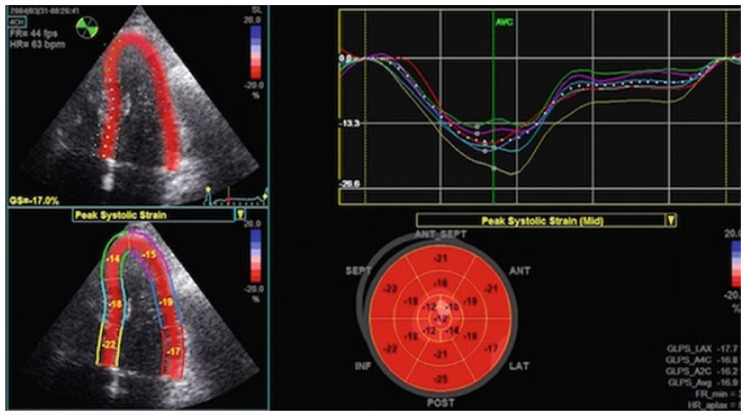
Left: LV 4-chambers view divided into six segments by the STE software https://www.gehealthcare.it/products/ultrasound/vivid/echopac/applications (accessed on 30 November 2023), each providing its own longitudinal strain value. Top: the curves of each segments’ longitudinal strain and the GLS’ curve. Bottom: the “bull’s eye”, the LV divided into seven segments, each with its own longitudinal strain; the GLS of each view and the average GLS is displayed on the right. LV: left ventricle; STE: speckle tracking echocardiography, GLS: global longitudinal strain.

STE is often used to assess LV function using several parameters, among which is the longitudinal strain, which is the cardiac deformation directed from the base to the apex: negative curves imply a reduction in the distance between the kernels caused by myocardial fiber shortening from the base to the apex [11]. Longitudinal strain analyzed in 4-, 2- and 3-chambers can assess both regional and global strain (normal range is from −17.2% to −27.7%) [11,12]. As a result, during systole and shortening of the speckle-to-speckle distance, longitudinal strain values are displayed as negative curves (Figure 1) [10,11,12].

**Figure 2:**
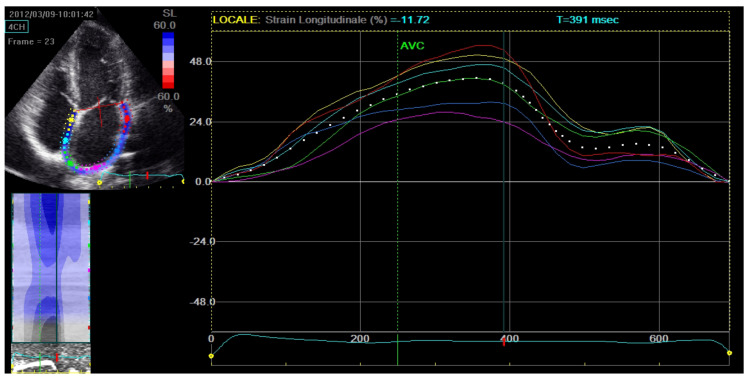
LA in a 4-chambers view divided into six segments by the STE software, each providing its own strain value. Top: the curves of the individual segments’ strain and the average curve, with the first peak representing PALS and the second peak PACS. LA: left atrium; STE: speckle tracking echocardiography, PALS: peak atrial longitudinal strain; PACS: peak atrial contraction strain.

Using the same STE principle, the technique can also be used to assess LA function during different phases of the cardiac cycle: reservoir (when LA receives blood from the pulmonary veins), conduit (when blood flows passively from LA to LV), and contraction (when the remaining blood is pumped out from the LA into LV) [13]. The STE software analyses images recorded in the apical 2- and 4-chamber views to provide a PALS value for each and the peak atrial contraction strain (PACS) (Figure 2) [13].

**Figure 3:**
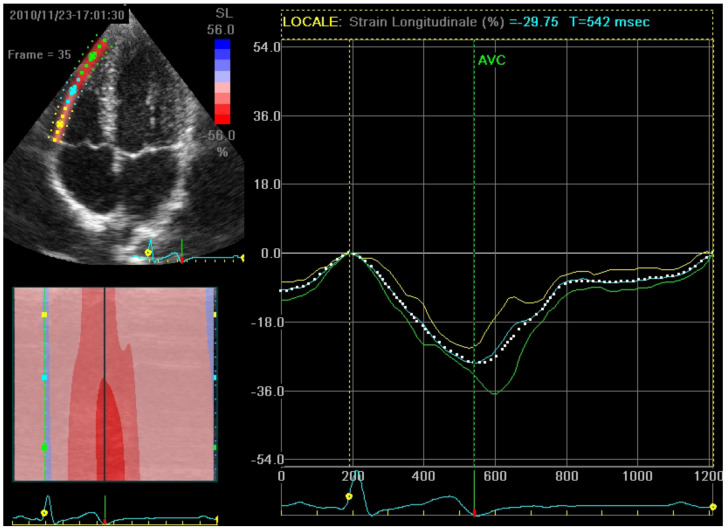
Top left: a RV free-wall in a 4-chambers view divided into three segments by the STE software, each providing its own longitudinal strain value. Right: the curve of each segment’s longitudinal strain and the average curve representing RVFWLS. RV: right ventricle; STE: speckle tracking echocardiography, RVFWLS: right ventricle free-wall longitudinal strain.

STE can also be used to assess right ventricular (RV) myocardial function from the apical 4-chamber view (Table 1). After defining the region of interest, which includes the RV free wall and the interventricular septum, the software calculates both free-wall longitudinal strain (RVFWLS, typically > −20%, Figure 3) and RV global longitudinal strain (GRVLS) [14].

## 3. Right Heart Catheterization

Right heart catheterization (RHC) is an invasive diagnostic procedure used in patients with HF to assess both left and right heart function, diagnose pulmonary hypertension (PH), analyze therapeutic response, and determine patients’ prognosis (Table 2). It is performed via either the internal jugular vein or the femoral vein using a Swan–Ganz catheter [15,16,17].

RHC measures right atrial pressure (RAP), which usually varies from 2 to 8 mmHg. The pressure waveform is typically defined by three peaks: the a wave, reflecting atrial contraction and RV filling, based on RV end-diastolic pressure (RV EDP); the c wave, representing tricuspid valve closure; and the v wave, coinciding with RV contraction. The three waves are separated by two falls, the x-descent and the y-descent, respectively [2,18].

After studying the RA the Swan–Ganz catheter is advanced to record RV pressures, both in systole and at end-diastole [2]. Proceeding further, the pressure tip manometer measures the pulmonary artery pressure (PAP), whose waveform is characterized by a fast pressure propagation from the RV, followed by a pressure fall at end-systole and a dicrotic notch reflecting pulmonary valve closure [15,16,17,18]. Blood flow, raised left atrial pressure (LAP), and pulmonary vascular resistance (PVR) all influence these measurements [2].

Pulmonary capillary wedge pressure (PCWP) is measured while the catheter is placed into a small pulmonary branch and it reflects effective LA pressure [2]. PCWP differentiates post-capillary PH (PCWP ≥ 15 mmHg) from pre-capillary PH (PCWP < 15 mmHg) [19].

LAP is measured by balloon occlusion of the distal pulmonary branches; it has a waveform similar to that of RA with a, c, and v waves, as well as negative x and y descents [2].

Finally, the LV pressure can also be studied, with its well-defined waveform which is identical to that of RV, but with higher systolic and diastolic pressures. LVEDP is used to measure preload and LV diastolic function [2].

RHC also allows calculation of hemodynamic parameters that can be used to determine myocardial function: the thermodilution technique and the Fick principle are both used to quantify cardiac output (CO) [2]. The thermodilution method involves injecting a 10–20 mL cold bolus into the catheter’s proximal part, while a thermistor in the distal end registers the differential temperature, and a specific software calculates the CO based on the injected temperature, volume, and blood specific gravity [20]. On the other hand, the Fick principle’s determination is based on the following equation:

$$VO_{2}=CO*1.34*Hb*(S_{AO2}-S_{VO2})$$

where VO_2_ is oxygen consumption, Hb is the hemoglobin blood concentration, and S_AO2_ and S_VO2_ represent, respectively, the arterial and mixed venous-blood oxygen saturation. Assuming an average VO_2_ value for every patient (usually 125 mL/min/m^2^), Hb, S_AO2_, and S_VO2_ can be directly measured, thus permitting CO determination [2,21].

RVSWI is a surrogate measurement of RV systolic function, being directly proportional to the stroke volume index (SVI). Values lower than 5 g × m^2^/beat, especially when paired with a PCWP > 20 mmHg and a V_O2_ < 14 mL/min/m^2^, are associated with increased mortality, the necessity for ventricular support device placement, and HTx at 1 year [2].

Finally, the pulmonary artery pulsatility index (PAPi) is a unique hemodynamic measure that is often used in the pre-operative evaluation of patients with advanced HF who require an LVAD or HTx [21] (Table 3). Most researchers have found an independent association between PAPi and survival [22].

The most common RHC complications are non-sustained ventricular and atrial tachycardia resulting from catheter contact with the chamber wall [2]; patients with left bundle branch block (LBBB) are more likely to experience intermittent complete atrioventricular block (AVB) [2]. Right bundle branch block (RBBB) occurs in 5% of patients [2]. Rare serious complications include RV rupture, pulmonary artery (PA) rupture, and RV infarction [23]. Minor complications, on the other hand, include venous spasm, bleeding, thrombophlebitis, atrial fibrillation, reversible LBBB or RBBB, and first- or second-degree AVB [2].

## 4. Speckle Tracking Echocardiography of Left Chambers

In 2020, the ESC described the HFpEF diagnostic algorithm (HFA-PEFF), which includes numerous Doppler echocardiographic measures related to LV filling pressures, such as indexed LA volume (LAVi), mitral annulus TDI, and E/e’ ratio, as well as pulmonary pressures, such as sPAP and retrograde tricuspid regurgitation peak velocity (TR_PV_) [24]. Novel echocardiographic measures, however, have been proved superior to traditional ones in assessing left heart function. PALS correlates strongly with LV filling pressures, particularly in patients with low EF, and it changes before LAVi [25]. Also, an inverse association between PALS and chronic HF patients’ quality of life measured by the Minnesota Living with Heart Failure Questionnaire (MLHFQ) has been shown [26].

Studies have demonstrated that STE has the ability to predict the presence of myocardial fibrosis (MF), with good accuracy [7]. Trials analyzing the presence of MF in hypertrophic cardiomyopathy (HCM) have shown how segments with MF detected by cardiac magnetic resonance (CMR) have lower longitudinal strain values [27] and extensive fibrosis, having reduced GLS [28]. Furthermore, in patients with myocarditis-related scars, segments with longitudinal strain < −12% have been associated with late gadolinium enhancement (LGE) on CMR, findings that are predictive of non-sustained ventricular tachycardias (NSVT) [29]. In the pediatric population, the technique was also able to identify oedema and sub-acute fibrosis in localized myocarditis despite a normal ejection fraction [30]. In patients with atrial fibrillation (AF) and end-stage HF, LA MF has been shown to be related to PALS, VO_2max_, NYHA class, LA stiffness, and E/e’ [7]. PALS, in particular, has a good correlation with NYHA class and VO_2max_, [31] and is a good predictor of MF [7]. These findings can be explained on the basis of the elevated LA pressure causing maladaptive remodeling including myocyte growth, hypertrophy, necrosis, and apoptosis. Furthermore, the fibroblast mitosis enhances extracellular matrix (ECM) with a switch into anaerobic metabolism, which leads to a reduction in the myocardial energy production [7]. Recently, PALS has been found to be accurate in reflecting LA reservoir function, with a capacity outperforming traditional measurement (LA volume and LV GLS) in predicting all-cause mortality and hospitalization [32].

The association between LA strain and cavity pressures was shown in a multicentric study of 322 patients with a mean LVEF 55%, where both PALS and PACS were found to be associated with LV filling pressures (LVFPs). The optimal cut-off for distinguishing normal from raised LVFP (PCWP > 12 mmHg) was 18% for PALS and 8% for PACS [33]. Similar results were obtained in 210 patients with LVEF >50%, where PALS accurately identified patients with increased PCWP > 15 mmHg compared to echocardiography and RHC, with an AUC of 0.76. Moreover, substituting TR peak velocity for PALS (<18%) in the 2016 ASE/EACVI algorithm led to 91% feasibility, 81% accuracy, and improved agreement with invasive measures [26]. Furthermore, the link between STE and LV pressures has been analyzed during stress tests, and showed PALS reduction in exercise-impaired HFrEF and HFpEF patients, which was associated with raised E/e′ [34]. A 2023 Chinese study found that, in patients with HCM, PALS measured at rest had the strongest association with METS ≤ 6.0 in treadmill stress echocardiography and had a good performance record in predicting different subtypes of HCM [35].

STE can also describe different systolic patterns of every myocardial layer: this feature, commonly known as multi-layer STE or layer-specific STE, has not been frequently used in the HF setting, but a few studies demonstrated how the epicardial layer GLS (GLS_Epi_) is a significant predictor of incident HF and cardiovascular diseases (CVDs) following ST-elevation myocardial infarction (STEMI) and also in the general male population [36,37] Furthermore, a 2019 trial demonstrated how the subendocardial-layer GLS (GLSEndo, −23.48 ± 2.70 vs. −23.02 ± 2.81; *p* = 0.043) and the GLSEndo/GLSEpi ratio (*p* = 0.034) were significantly associated with dyspnea, contrary to other echocardiographic variables [38].

In end-stage HF, LVEF has failed to predict clinical outcomes in the short- and long-term. In contrast, LVGCS outperformed LVGLS in predicting long-term mortality and future clinical events, with higher sensitivity and specificity [9]. This is most likely because the fibers in the LV mid-wall (associated with circumferential strain) have greater intrinsic contractile activity than other myocardial fibers [11]. In contrast, in HFrEF patients, there is a modest relationship between transverse LV function and MF, but a significant association with GLS, which proved to have a stronger predictive value than other echocardiographic measures [39]. A 2023 meta-analysis evidenced how this parameter was strongly related with peak VO_2_ measured during the cardiopulmonary exercise test compared with LV EF, and is even linked with cardiorespiratory fitness indices in HFrEF [40].

Recently, STE has also been applied to 3D real-time echocardiography (34 RTE), producing a 3D-STE technique. This method has proved accurate in left ventricular systolic function [10]. Several trials investigated the role of both 2D-STE GLS and 3D-STE GLS in HF, and have shown the latter to be a powerful independent predictor of MACE in asymptomatic aortic stenosis (2D GLS −14.7 ± 3.3 vs. −16.3 ± 3.3, *p* = 0.0168; 3D GLS −13.5 ± 2.5 vs. −16.1 ± 2.4, *p* < 0.0001) [41], and to be superior in predicting STEMI patients’ prognosis [42]. 3D-STE has also been applied to assess atrial function in the form of LA Emptying Fraction (LAEmpF), which proved an independent predictor of hospitalization in HF patients, thus providing higher prognostic power in future MACEs than all conventional 2D-based parameters (AUC = 0.82, *p* < 0.0001; cut-off value < 0.420) [43].

## 5. Speckle Tracking Echocardiography of Right Heart Chambers

RV remodeling limits longitudinal performance while increasing transverse function by reducing the circumferential fibers of the outer myocardial layer. This anatomical fact explains why RVFWLS is a sensitive measure for diagnosing RV dysfunction [9]. End-stage HF patients exhibit a significant relationship between RVFWLS and histologically confirmed MF, with RVFWLS being the primary determinant and the main predictor of MF [44]. This parameter has been shown to be the most reliable diagnostic tool for detecting severe MF in patients with extensive RV fibrosis [44]. The RV free-wall function itself has been proved to be the most accurate parameter that predicts exercise capacity and clinical outcome in patients with dilated cardiomyopathy (DCM) [45].

In patients with PH, RV strain can accurately predict clinical outcome, being associated with increased risk of all-cause mortality [46]. Ischemic patients with low RVFWLS values have been shown to have worse survival [12], having been proved to have profound transmural MF, particularly in AMI [46].

Also, RVFWLS is linked with structural cardiopathy risk of hospitalization, which is worse with increasing NYHA class and higher NT pro–B-type natriuretic peptide [47].

RV strain has been evaluated in advanced-HF patients, especially in the pre-operative LVAD evaluation. Standard echocardiographic indices, such as tricuspid annulus s’ and tricuspid annular plane systolic excursion (TAPSE), do not have a strong relationship with clinical prognosis [11]. On the other hand, recent studies have shown good correlation between pre-operative RVFWLS and the development of RV failure after LVAD implantation. The first study was published in 2012, when Grant et al. demonstrated poor prognosis and RVF in patients with RVFWLS lower than −12% [48]. More recent studies and a meta-analysis confirmed these results and also showed how RVFWLS is the best predictor of RV failure (RVF) following LVAD implantation, and an independent risk factor for RVF development such as RVSWI [49]. Furthermore, a reduced value has been shown after surgery, despite other RV echocardiographic parameters remaining unchanged during the stress test, with the pump speed optimized for resting conditions [50].

Three-dimensional STE has also been applied to RV, not only for assessing systolic function but also for determining its volumes [51]. Meng et al. showed 3D-STE parameters to have similar predictive value as 2D-STE indices in patients with HFpEF (3D STE RVFWLS HR 5.73; 95% CI 2.77–11.85; *p* < 0.001; 2D STE RVFWLS R 3.17; 95% CI 1.54–6.53; *p* = 0.002) [51]. Moreover, 3D-STE indices have proved to have comparable predictive ability for adverse cardiac events [52] and mortality in patients with PH [53].

## 6. Discussion

In HF patients, there is significant relationship between LA strain, MF and clinical outcome. PALS is also related to both LV filling pressures, and is a good measure of diastolic dysfunction and myocardial VO_2_, which is a HF fundamental survival marker. Furthermore, LA strain allows detailed analysis of heart chambers’ histopathologic status and metabolic activity, with the latter being a key prognostic marker in patients with advanced HF.

**Figure 4:**
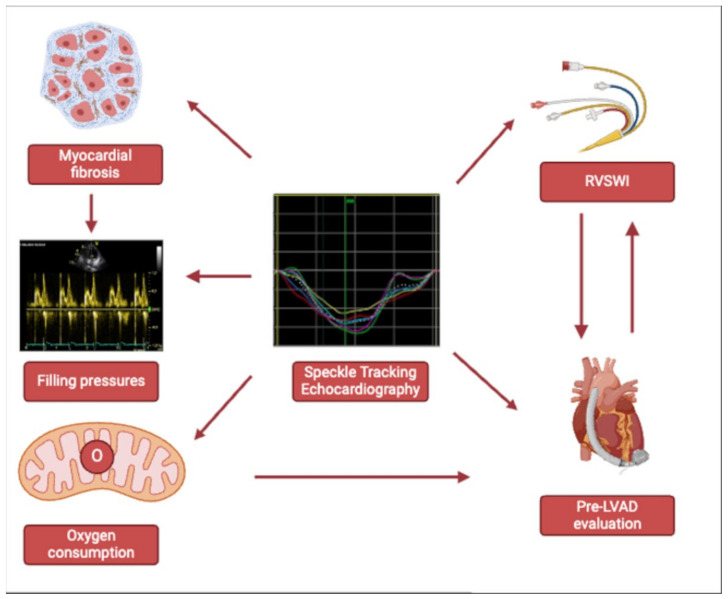
The STE network for advanced-HF evaluation and treatment. RVSWI: right ventricle stroke work index; LVAD: left ventricular assist device.

Studies have shown that STE has significant value for patient management and in guiding towards optimum treatment strategies. RVFWLS is not only related to RVSWI but also to development of RV failure, thus making it critical in the pre-LVAD implantation evaluation. Because of these relationships and the higher pace at which these parameters change with changes in medical condition, it has become evident that STE plays an essential role in the evaluation of both early and advanced HF, allowing not only the prevention of major myocardial damage but also treating it with the most appropriate therapy (Figure 4).

On the other hand, STE has significant limitations. Firstly, it requires an optimal echocardiographic window, which is not always possible because of either the patient’s clinical disease (e.g., COPD and interstitial fibrosis) and/or the setting (Intensive Care Units) where the echo study is performed. Moreover, optimum recording and analysis requires ECG gating with regular rhythm, which is not feasible in patients with atrial fibrillation (AF). Furthermore, post-operative transthoracic echocardiographic assessment could be limited because of the need for monitoring wires and management tubes. Even after hospital discharge, in patients with LVAD, the limited image acquisition remains because of the electromagnetic waves interfering with the ultrasound. The available studies of such issues are mostly single-centered with only one meta-analysis. Also, there is no established consensus regarding the RVFWLS cut-off below which LVAD implantation is contraindicated due to the RVF risk.

Three-dimensional STE suffers from the same 2D-STE’s limitations, requiring an optimal acoustic window and high temporal resolution. Also, 3D-STE requires multi-beat acquisition, thus limiting its use in arrhythmia patients. Moreover, the software does not permit manual ROI position adjustment and does not have an automatic validation of STE. Finally, with the optimal frame rate of 35–50 vps for 3D-STE, frame rates below 18 vps would lead to significant underestimation of strain magnitude.

The above issues highlight the fact that the STE is operator-dependent. In addition, the above limitations have contributed to the modest reproducibility of STE measurements, even when using the same echocardiographic system. Finally, the limitation of measurement reproducibility among different manufactures remains outstanding.

## 7. Conclusions

There is no consensus regarding the use of STE in patients with advanced HF, especially in pre-operative LVAD evaluation, and the limitation of an adequate acoustic window does not permit its reliable use in all patients. For STE to become valuable for routine use, stronger evidence for its accurate feasibility is required, as well as cut-off values for patients with advanced heart failure.

## Figures and Tables

**Table 1:** STE normal range for different cardiac chambers [10,11,12,13,14].

Parameter	Reference Values (%)
Left ventricle	
GLS	−17.2–−27.7
Left atrium	
PALS	42.3–52.4 age 20–40
	35.4–46.1 age 40–60
	30.9–41.9 age > 60
PACS	11.9–19.0 age 20–40
	13.2–19.6 age 40–60
	13.6–21.4 age > 60
Right ventricle	
RVFWS	>−20

STE: speckle tracking echocardiography; GLS: global longitudinal strain; PALS: left atrium strain reservoir; PACS: left atrium strain conduit phase; RVFWS: right ventricle free-wall strain.

**Table 2:** Current indications for RHC [2].

HTx Check List
Diagnosis and differential diagnosis for PH
Fulminant myocarditis
Peripartum cardiomyopathy
Differential diagnosis for sepsis
ADHF requiring inotropic, vasopressor, and vasodilator therapy
Cardiogenic shock
Discordant left- and right-ventricular dysfunction

RHC: right heart catheterization; HTx: heart transplantation; PH: pulmonary hypertension; ADHF: acute decompensated heart failure.

**Table 3:** RHC main parameter reference values [2,21].

Parameter	Reference Values
Right atrium	
Mean RAP	2–8 mmHg
Right ventricle	
RVESP	17–32 mmHg
RVEDP	2–8 mmHg
Pulmonary artery	
mPAP	10–21 mmHg
sPAP	17–32 mmHg
dPAP	4–15 mmHg
PCWP	2–8 mmHg
Left atrium	
Mean LAP	6–12 mmHg
Left ventricle	
LVESP	90–140 mmHg
LVEDP	5–12 mmHg
Derived parameters	
CO	2.5–4.5 mL/min/m^2^
PVR	<2 WU
RVSWI	5–10 g*m^2^/beat
PAPi	<0.9 in RV infarction <1.85 in patients undergoing LVAD implantation <3.65 in patients with advanced HF

RAP: right atrium pressure; RVESP: right ventricle end-systolic pressure; RVEDP: right ventricle end-diastolic pressure; mPAP: mean pulmonary artery pressure; sPAP: systolic pulmonary artery pressure; dPAP: diastolic pulmonary artery pressure; PCWP: pulmonary capillary wedge pressure; LAP: left atrium pressure; LVESP: left ventricle end-systolic pressure; LVEDP: left ventricle end-diastolic pressure; CO: cardiac output; PVR: pulmonary vascular resistance; RVSWI: right ventricle stroke work index; PAPi: pulmonary artery pulsatility index; LVAD: left ventricular assist device; HF: heart failure.

## Data Availability

The publications can be read using the Pubmed platform, the journals and the books mentioned in the references.
